# An Altered Pattern of Myocardial Histopathological and Molecular Changes Underlies the Different Characteristics of Type-1 and Type-2 Diabetic Cardiac Dysfunction

**DOI:** 10.1155/2015/728741

**Published:** 2015-01-05

**Authors:** Tamás Radovits, Sevil Korkmaz, Csaba Mátyás, Attila Oláh, Balázs Tamás Németh, Szabolcs Páli, Kristóf Hirschberg, Alina Zubarevich, Patricia Neh Gwanmesia, Shiliang Li, Sivakkanan Loganathan, Enikő Barnucz, Béla Merkely, Gábor Szabó

**Affiliations:** ^1^Department of Cardiac Surgery, University of Heidelberg, INF 326 OG 2, 69120 Heidelberg, Germany; ^2^Heart and Vascular Center, Semmelweis University, Városmajor u. 68, 1122 Budapest, Hungary

## Abstract

Increasing evidence suggests that both types of diabetes mellitus (DM) lead to cardiac structural and functional changes. In this study we investigated and compared functional characteristics and underlying subcellular pathological features in rat models of type-1 and type-2 diabetic cardiomyopathy. Type-1 DM was induced by streptozotocin. For type-2 DM, Zucker Diabetic Fatty (ZDF) rats were used. Left ventricular pressure-volume analysis was performed to assess cardiac function. Myocardial nitrotyrosine immunohistochemistry, TUNEL assay, hematoxylin-eosin, and Masson's trichrome staining were performed. mRNA and protein expression were quantified by qRT-PCR and Western blot. Marked systolic dysfunction in type-1 DM was associated with severe nitrooxidative stress, apoptosis, and fibrosis. These pathological features were less pronounced or absent, while cardiomyocyte hypertrophy was comparable in type-2 DM, which was associated with unaltered systolic function and increased diastolic stiffness. mRNA-expression of hypertrophy markers c-fos, c-jun, and *β*-MHC, as well as pro-apoptotic caspase-12, was elevated in type-1, while it remained unaltered or only slightly increased in type-2 DM. Expression of the profibrotic TGF-*β*_1_ was upregulated in type-1 and showed a decrease in type-2 DM. We compared type-1 and type-2 diabetic cardiomyopathy in standard rat models and described an altered pattern of key pathophysiological features in the diabetic heart and corresponding functional consequences.

## 1. Introduction

Depending on its pathomechanism, diabetes can be classified into two main types, namely, type-1 and type-2 diabetes mellitus (DM). It is a well-known phenomenon that type-2 diabetes, most common form of diabetes, is dominated by hyperinsulinaemia, hyperglycaemia, and dyslipidaemia, while type-1 diabetes, due to the autoimmune-mediated destruction of the *β* cells of the Langerhans islets in the pancreas, is characterized by insulinopenia, hyperglycaemia, and a largely unaffected lipid profile. Furthermore, the onset of type-1 diabetes is acute, unlike that of type-2 diabetes, which is characterized by a period of insulin resistance, hyperinsulinaemia, and euglycaemia preceding the onset of hyperglycaemia. Although increased atherosclerosis of the coronary arteries is thought to be responsible for a major portion of the mortality in diabetic patients, there is evidence supporting the existence of diabetes-induced direct structural and functional alterations at the level of the myocardium [1]. The development of diabetic cardiomyopathy has been described in both types of DM, also in the absence of coronary artery disease and hypertension, and is suggested to be a consequence of altered cellular metabolism of the myocardium, which increases the risk of heart failure [2].

For decades, experimental studies on diabetic cardiomyopathy were performed on streptozotocin- (STZ-) induced type-1-diabetic rodents, the most widely used, “standard” animal model of DM. Nevertheless, the majority of diabetic patients have type-2 diabetes with a different pathophysiological background. Therefore a number of animal models of type-2 DM have been developed and become object of intensive cardiovascular investigations recent years [3–5].

Our research group previously provided a detailed hemodynamic characterisation of rat models of type-1 (STZ-induced) and type-2 (Zucker Diabetic Fatty rats) DM by the sophisticated method of left ventricular (LV) pressure-volume (P-V) analysis. Different characteristics of type-1 and type-2 diabetic cardiac dysfunction could be demonstrated: decreased systolic performance and delayed relaxation, mainly in type-1 DM; accompanied by increased diastolic stiffness of the heart, more remarkably in type-2 DM [6].

The underlying pathophysiological features and subcellular mechanisms responsible for the development of diabetic cardiomyopathy are not completely understood. Metabolic abnormalities and overproduction of reactive oxygen and nitrogen species may trigger various intracellular pathways and alter myocardial expression of different genes. Increased nitrooxidative stress, cardiomyocyte hypertrophy, profibrotic signalling, and myocardial remodelling along with apoptotic processes have been reported to play a critical role in the development of cardiomyopathy in both types of DM [7–9].

To what extent these characteristic molecular and cellular changes develop in type-1 versus type-2 DM and how they correspond with the described differences in several aspects of cardiac dysfunction also remain to be elucidated. Therefore, in the present study we aimed at describing myocardial histological and molecular changes which may be involved in different characteristics of LV dysfunction in the well-established rat models of type-1 and type-2 DM [6, 10].

## 2. Materials and Methods

### 2.1. Animals

Male 12-week-old Sprague-Dawley (SD; Charles River, Sulzfeld, Germany), Zucker Diabetic Fatty (ZDF), and ZDF lean rats (Charles River, Kingston, NY) were housed in a room at 22 ± 2°C under 12-h light/dark cycles. Sprague-Dawley rats were fed a standard laboratory rat diet and ZDF rats a diet of Purina 5008 as recommended by the supplier and water ad libitum. The rats were acclimatized for at least 1 week before the experiments. All animals received humane care in compliance with the Principles of Laboratory Animal Care formulated by the National Society for Medical Research and the* Guide for the Care and Use of Laboratory Animals* prepared by the Institute of Laboratory Animal Resources and published by the National Institutes of Health (NIH Publication no. 86-23, revised 1996). This investigation was reviewed and approved by the appropriate institutional review committee.

### 2.2. Rat Model of Type-1 DM

Type-1 DM was induced in rats (*n* = 8) with a single injection of streptozotocin (STZ, 60 mg/kg, i.p.) freshly dissolved in 0.1 M citrate buffer, pH = 4.5. Vehicle-treated animals served as nondiabetic controls (STZ control group, *n* = 10). Seventy-two hours after STZ injection, a drop of blood was collected from the tail vein, and the blood glucose concentration was determined using a digital blood glucose meter and test strips (Accu-Chek Sensor, Roche, Mannheim, Germany). Animals with a random blood glucose level of >15 mM were considered as diabetic and were included into the study (STZ-induced diabetic group). According to the pathophysiology and disease characteristics of type-1 DM, based on literature data and results of our previous studies, experiments on the rats were performed 8 weeks after the confirmation of diabetes [6].

### 2.3. Rat Model of Type-2 DM

The ZDF rat is an inbred rat model that through genetic mutation and a special diet develops type-2 diabetes and related complications. Homozygous recessive males (*fa*/*fa*) develop obesity, fasting hyperglycemia, and type-2 diabetes (ZDF Diabetic group, *n* = 8). Homozygous dominant (+/+) and heterozygous (*fa*/+) lean genotypes remain normoglycemic (ZDF Lean group, *n* = 9). According to the pathophysiology and disease characteristics of type-2 DM, based on literature data and results of our previous studies, experiments on the rats were performed at the age of 30–32 weeks [6].

### 2.4. Blood and Urine Glucose Measurements

Before the hemodynamic measurements, blood samples were collected from the tail vein. Urine samples were collected by punctuation of the urine bladder after hemodynamic measurements had been completed. Blood and urine glucose levels were determined by a digital blood glucose meter and test strips (Accu-Chek Sensor, Roche).

### 2.5. Hemodynamic Measurements

Rats were anesthetized with a mixture of ketamine (100 mg/kg) and xylazine (3 mg/kg) i.p., tracheotomized, and intubated to facilitate breathing. The animals were placed on controlled heating pads and core temperature measured via a rectal probe was maintained at 37°C. A polyethylene catheter was inserted into the left external jugular vein for fluid administration. A 2F microtip P-V catheter (SPR-838, Millar Instruments, Houston, TX, USA) was inserted into the right carotid artery and advanced into the ascending aorta. After stabilization for 5 min, arterial blood pressure was recorded. After that, the catheter was advanced into the left ventricle under pressure control. After stabilization for 5 min, the signals were continuously recorded at a sampling rate of 1000/s using a P-V conductance system (MPVS-400, Millar Instruments, Houston, TX, USA), stored, and displayed on a personal computer by the PowerLab Chart5 Software System (ADInstruments Inc., Colorado Springs, CO, USA). In addition, P-V-loops at different preloads were registered by transiently compressing the inferior caval vein (reducing preload) under the diaphragm with a cotton-tipped applicator. With the help of a special P-V analysis program (PVAN, Millar Instruments, Houston, TX, USA) ejection fraction (EF) and the slope of the LV end-systolic P-V relationship (ESPVR) (according to the parabolic curvilinear model) were calculated as load-dependent and -independent indices of LV contractility, respectively. LV end-diastolic pressure (LVEDP) and the slope of the LV end-diastolic P-V relationship (EDPVR) were calculated as reliable indexes of LV stiffness [11].

The in vivo and in vitro volume calibrations of the conductance system were performed as described previously [11].

### 2.6. Histopathology

Hearts were fixed in buffered paraformaldehyde solution (4%) and embedded in paraffin. Then, 5-*μ*m thick sections were placed on adhesive slides and stained with hematoxylin-eosin and Masson's trichrome. Light microscopic examination was performed with a Zeiss microscope (Axio Observer.Z1, Carl Zeiss, Jena, Germany) and digital images were captured using an imaging software (QCapture Pro 6.0, QImaging, Canada). The transverse transnuclear widths of randomly selected cardiomyocytes were measured after calibrating the system. The mean value of 100 LV cardiomyocytes represents each sample. The amount of myocardial collagen was determined by semiquantitative morphometry scoring of Masson's trichrome-stained sections by one blinded observer as follows: 0: absent, 1: slight, 2: moderate, and 3: intense. The mean value of twenty randomly selected visual fields (magnification 400x) of free LV wall represents each sample.

### 2.7. Terminal Deoxynucleotidyl Transferase-Mediated dUTP Nick-End Labeling (TUNEL) Assay

The detection was carried out using a commercial kit following the protocol provided by the manufacturer (Chemicon International, Temecula, CA, USA). Rehydrated sections were treated with 20 *μ*g/mL DNase-free Proteinase-K (Sigma-Aldrich, Germany) to retrieve antigenic epitopes, followed by 3% hydrogen peroxide to quench endogenous peroxidase activity. Free 3′-OH termini were labeled with digoxigenin-dUTP for 1 h at 37°C utilizing a terminal deoxynucleotidyl transferase reaction mixture (Chemicon International, Temecula, CA, USA). Incorporated digoxigenin-conjugated nucleotides were detected using a horseradish peroxidase conjugated anti-digoxigenin antibody and 3,3′-diaminobenzidine. Sections were counterstained with Gill's hematoxylin. Dehydrated sections were cleared in xylene, mounted with Permount (Fischer Scientific, Germany), and coverslips were applied. Based on the intensity and distribution of labelling, semiquantitative histomorphological assessment was performed using conventional microscopy. For assessment of TUNEL-labeled cells, the number of TUNEL positive and total cardiomyocyte nuclei was counted (four microscopic examination fields characterizing each specimen), and an average percent value was calculated. The evaluation was conducted by an investigator blinded to the study groups. Data were expressed as fold change to the mean value of the corresponding nondiabetic control group.

### 2.8. Nitrotyrosine Immunohistochemical Staining

According to previously described methods [12] we performed immunohistochemical staining for nitrotyrosine, a marker of nitrooxidative stress. Semiquantitative histomorphological assessment was performed based on the intensity and distribution of labelling using conventional microscopy. After initially evaluating all corresponding tissue sections (magnification 200x), the tissue section with the most intense labelling signals was used as a reference for maximum labelling intensity. Each specimen was characterized with the average of 4 adjacent fields. Nitrotyrosine levels were scored as follows: 0: complete absence of immunoreactivity, 1: weak area of staining, 2: intermediate staining, and 3: extensive staining. Using the Cell^∧^A software (Olympus Soft Imaging Solutions GmbH, Germany), we measured the area of the objects in each class in each field, assigned an area score (1 ≤ 10% positive cells, 2 = 11–50% positive cells, 3 = 51–80% positive cells, and 4 ≥ 80% positive cells), and calculated an average score for the whole picture (intensity score multiplied by area score, 0–12). The evaluation was conducted by an investigator blinded to the experimental groups.

### 2.9. Quantitative Real Time Polymerase Chain Reaction (PCR)

Total RNA was extracted from LV myocardial samples using the RNeasy Fibrous Tissue Mini Kit (Qiagen, Hilden, Germany) according to the manufacturer's instructions. RNA concentration and purity were determined photometrically (260, 280, and 230 nm). Reverse transcription was performed with the QuantiTect Reverse Transcription Kit (Qiagen, Hilden, Germany) using 400 ng RNA in a volume of 20 *μ*L. Quantitative real-time PCRs were performed on the StepOnePlus Real-Time PCR System with TaqMan Universal PCR MasterMix and TaqMan Gene Expression Assays (Applied Biosystems, Foster City, USA) in case of endogenous antioxidant genes, or on the LightCycler480 system with the LightCycler480 Probes Master and Universal Probe Library (UPL) probes (Roche, Mannheim, Germany) in case of all other genes. Efficiency of the PCR reaction was confirmed with standard curve analysis. Sample quantifications were normalized to glyceraldehyde-3-phosphate dehydrogenase (GAPDH) expression in case of endogenous antioxidants and to *β*-actin expression in case of all other genes by using a pool of all cDNAs from the control groups (positive calibrator). Identification numbers of TaqMan Gene Expression Assays (endogenous antioxidant gene targets) as well as UPL probes and sequences of primers used (TIB Molbiol, Berlin, Germany) (all other gene targets) are represented in Table 1. Evaluation was performed with the StepOne Software 2.2.2 (Applied Biosystems, Foster City, USA) and LightCycler 480 SW 1.5 software (Roche, Mannheim, Germany), respectively.

### 2.10. Western Blot

Myocardial proteins were extracted into a solution containing 8 M urea, 5 mM EDTA, 0.002% Trasylol, 0.05 mM PMSF, 0.003% Triton X-100 containing protease inhibitors (Roche, Mannheim, Germany). Protein concentration was determined by a commercial kit according to the manufacturer's protocol (BCA protein assay kit; Thermo Scientific, Rockford, USA). Total protein homogenates (30 *μ*g) were denatured, separated on SDS-PAGE gradient gels (Invitrogen, Darmstadt, Germany), and transferred to PVDF membrane (Invitrogen, Darmstadt, Germany). The blots were probed with an antibody specific to TGF-*β*_1_ (1 : 1000, Santa Cruz Biotechnology, Heidelberg, Germany). The immunoreactive protein bands were developed using Enhanced Chemiluminescence system (ECL Plus, PerkinElmer, Rodgau-Juegesheim, Germany) and measured by an imaging densitometer with image analysis software (Image J. NIH, Bethesda, MD, USA). Densitometric data of each sample was normalized to the mean value of the corresponding nondiabetic control group.

### 2.11. Statistical Analysis

All data are expressed as means ± standard error of the mean (SEM). An unpaired two-sided Student's* t*-test was used to compare parameters of diabetic and control rats in both models. A value of *P* < 0.05 was considered statistically significant.

## 3. Results

### 3.1. Heart Weight, Body Weight, and Glucose Levels

Compared with the corresponding control group, DM was associated with decreased body weight and increased blood and urine glucose levels in both models (Table 2). Blood glucose levels did not significantly differ between the two diabetic groups. Heart weight to body weight ratio showed a marked tendency towards increased values in the diabetic animals of the type-2 DM model, while it reached a statistical significance in the type-1 DM model (Table 2).

### 3.2. Cardiac Contractility and Ventricular Stiffness

**Figure 1:**
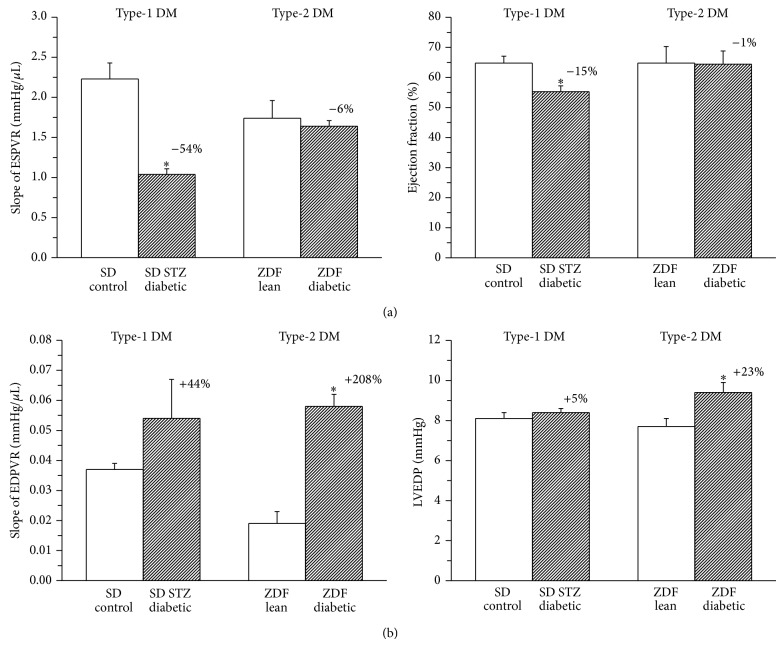
Different characteristics of cardiac dysfunction in type-1 and type-2 diabetes mellitus (DM) models. Left ventricular contractility indices (a): slope of end systolic pressure-volume relationship (ESPVR) and ejection fraction (EF) in the groups of SD control, STZ-induced diabetic SD, ZDF lean and ZDF diabetic rats. Parameters of left ventricular stiffness (b): slope of end-diastolic pressure-volume relationship (EDPVR) and end-diastolic pressure (LVEDP) in the groups of SD control, STZ-induced diabetic SD, Zucker Diabetic Fatty (ZDF) lean and ZDF diabetic rats. Bar graphs represent mean ± SEM. Percent changes between diabetic and corresponding nondiabetic control groups are indicated in both DM models. ^*^: *P* < 0.05 versus the corresponding nondiabetic group.

Mean arterial pressure did not significantly differ between diabetic rats and corresponding controls (data not shown). P-V loop derived load-dependent and -independent contractility parameters (EF and slope of ESPVR) were significantly lower in STZ-induced diabetic animals compared to nondiabetic controls, suggesting impaired systolic performance in type-1 DM. In contrast, compared with the corresponding control, type-2 diabetic ZDF rats showed unaltered LV contractility (Figure 1(a)).

LVEDP and the slope of EDPVR were significantly increased in diabetic animals of the type-2 DM model, indicating a marked increase in end-diastolic stiffness. These changes could be observed only to a lower extent in type-1 diabetic rats (Figure 1(b)).

### 3.3. Histopathology

**Figure 2:**
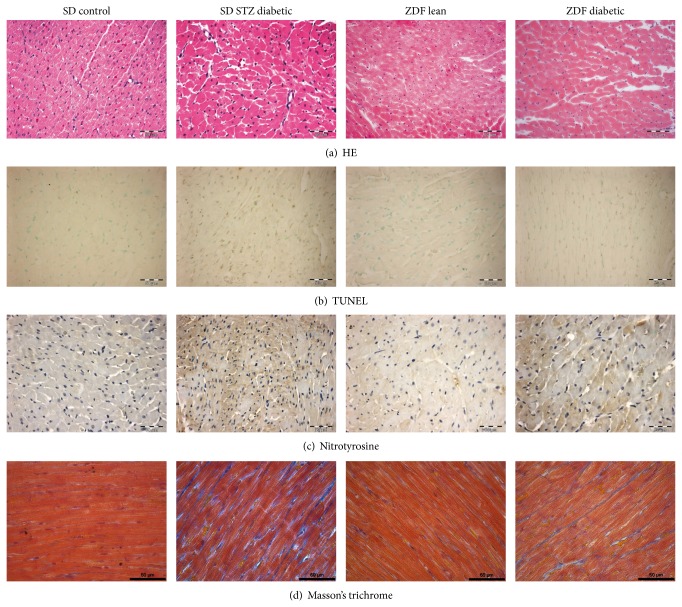
Characteristic histopathological changes of the myocardium in type-1 and type-2 diabetes mellitus (DM) models. Representative images of conventional histopathological examination (hematoxylin-eosin (HE) staining; scale bar: 50 μm, (a)), TUNEL staining for DNA breaks and apoptosis (brown cell nuclei, scale bar: 50 μm, (b)), immunohistochemistry for the nitrooxidative stress marker nitrotyrosine (brown staining, scale bar: 50 μm, (c)) and Masson's trichrome staining for fibrosis detection (blue staining, scale bar: 60 μm, (d)) of left ventricular myocardium in the groups of SD control, streptozotocin- (STZ-) induced diabetic SD, ZDF lean and ZDF diabetic rats.

**Figure 3:**
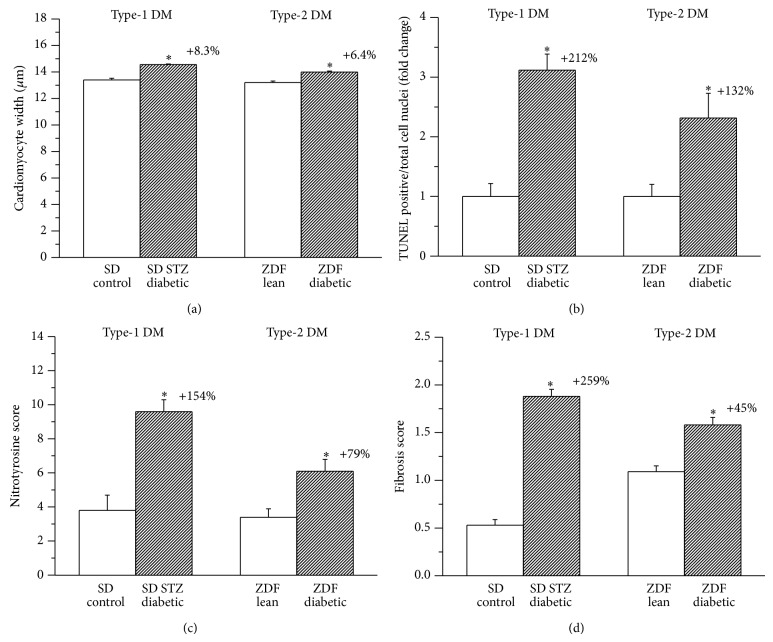
Quantification of histopathological changes of the myocardium in type-1 and type-2 diabetes mellitus (DM) models. Mean cardiomyocyte width as marker of myocardial hypertrophy (a), fold change in the number of TUNEL-positive/total cardiomyocyte nuclei (b), semiquantitative histomorphological scoring of nitrotyrosine immunostaining (c), and Masson's trichrome fibrosis staining (d) in the groups of SD control, streptozotocin- (STZ-) induced diabetic SD, Zucker Diabetic Fatty (ZDF) lean, and ZDF diabetic rats. Bar graphs represent mean ± SEM. Percent changes between diabetic and corresponding nondiabetic control groups are indicated in both DM models. ^*^: *P* < 0.05 versus the corresponding nondiabetic group.

The characteristics of diabetic cardiomyopathy were found in LV sections of the diabetic groups with hematoxylin-eosin staining (Figure 2(a)). Disarray and collapse of myofibers and myocardial degeneration could be observed in the LV myocardium of diabetic rats in both models. Mean cardiomyocyte width, as a marker of cardiomyocyte hypertrophy was significantly increased in both diabetic groups when compared to the corresponding controls (Figure 3(a)).

### 3.4. DNA Strand Breaks and Nitrooxidative Stress

Compared with the corresponding controls, diabetic hearts were associated with increased density of TUNEL-positive nuclei, indicating DNA fragmentation (Figures 2(b) and 3(b)) and increased immunoreactivity against the nitrooxidative stress marker nitrotyrosine, as evidenced by increased brown staining (Figure 2(c)). Moreover, both TUNEL positivity and nitrooxidative stress were found to be more pronounced in type-1 when compared with the type-2 DM group (Figure 3(c)).

**Figure 4:**
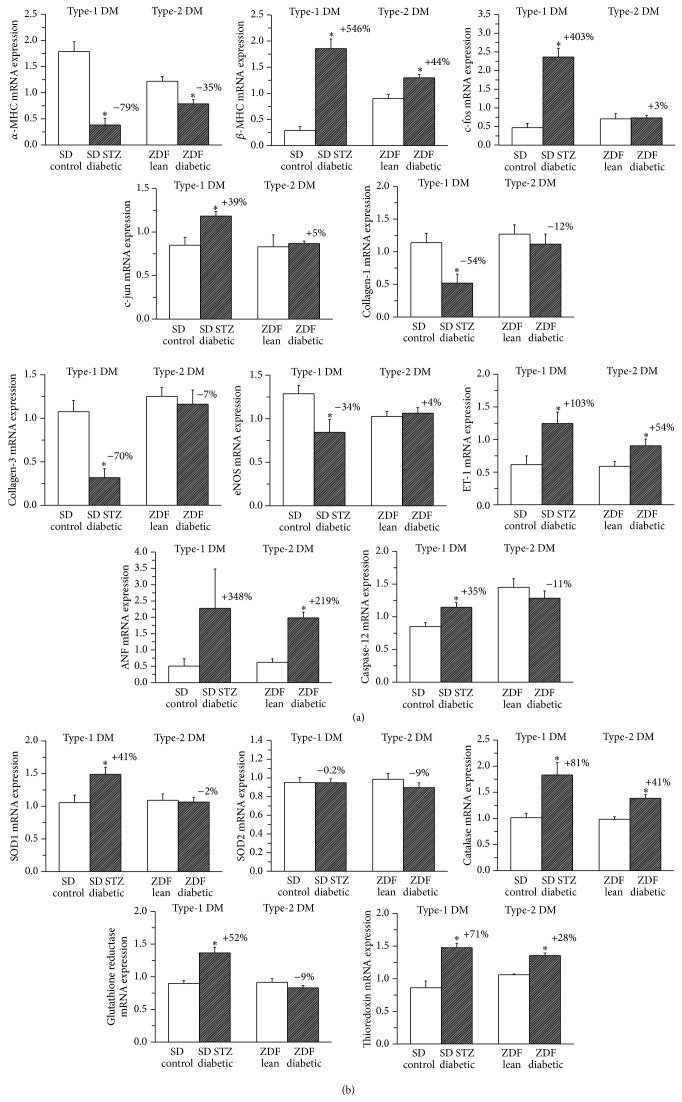
Characteristic changes in myocardial expression of genes involved in the development of diabetic cardiomyopathy (a) and genes of endogenous antioxidants (b) in type-1 and type-2 diabetes mellitus (DM) models. Relative mRNA expression for (a) hypertrophy markers α myosin heavy chain (MHC), β-MHC, early genes of the hypertrophic transcriptional program c-fos and c-jun; extracellular matrix components collagens 1 and 3, vascular endothelial marker endothelial nitric oxide synthase (eNOS) and endothelin-1 (ET-1); atrial natriuretic factor (ANF) and proapoptotic caspase-12, as well as for (b) endogenous antioxidants superoxide dismutase 1 (SOD1) and 2 (SOD2), catalase, glutathione reductase and thioredoxin in the groups of SD control, streptozotocin- (STZ-) induced diabetic SD, Zucker Diabetic Fatty (ZDF) lean and ZDF diabetic rats. Bar graphs represent mean ± SEM. Percent changes between diabetic and corresponding nondiabetic control groups are indicated in both DM models. ^*^: *P* < 0.05 versus the corresponding nondiabetic group.

Quantitative real-time PCR from LV myocardial RNA extracts revealed that mRNA-expression for endogenous antioxidants cytosolic superoxide dismutase 1 (SOD1), catalase, glutathione reductase (GSR), and thioredoxin was significantly upregulated in type-1 DM, while these changes were less pronounced or absent in the type-2 DM model. mRNA expression for the mitochondrial superoxide dismutase 2 (SOD2) was not altered in any groups studied. (Figure 4).

### 3.5. Myocardial Fibrosis

Myocardial fibrotic remodelling, as reflected by Masson's trichrome staining of myocardial sections, was significantly more pronounced in the type-1 DM model, when compared to type-2 DM (Figure 2(d)). Semiquantitative scoring of the staining showed a marked difference in LV fibrosis between the two diabetic models (Figure 3(d)).

### 3.6. Myocardial Expression of Genes Involved in the Development of Diabetic Cardiomyopathy

Quantitative real-time PCR from LV myocardial RNA extracts revealed that mRNA expression for *α*-myosin heavy chain (*α*-MHC) was significantly decreased whereas *β*-MHC and endothelin-1 mRNA-levels were increased in both diabetic groups compared to corresponding controls (Figure 4). Whereas c-fos, c-jun, and caspase-12 mRNA-expression was significantly upregulated, collagen-1, collagen-3, and endothelial NOS mRNA-levels were downregulated only in type-1 DM when compared with the control group (Figure 4). Atrial natriuretic factor (ANF) mRNA expression was upregulated in both types of DM, but the change reached the level of statistical significance only in the type-2 model (Figure 4). The gene expression changes for *α*-MHC, *β*-MHC, c-fos, c-jun, caspase-12, collagen-1, collagen-3, and endothelial NOS were significantly more pronounced in type-1 when compared to the type-2 DM group (Figure 4).

### 3.7. Western Blot Analysis of TGF-*β*_1_

**Figure 5:**
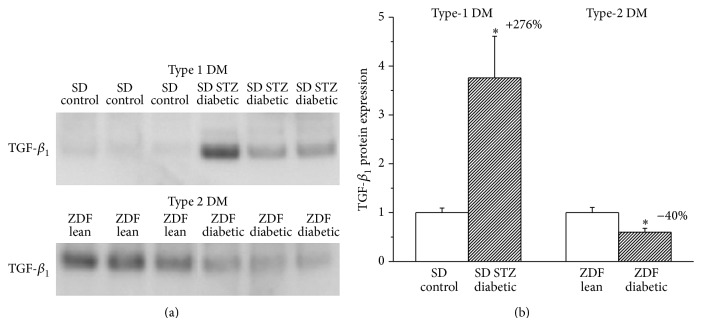
Changes of the myocardial protein expression of transforming growth factor (TGF)-*β*_1_ in type-1 and type-2 diabetes mellitus (DM) models. Representative immunoblot analysis for the profibrotic mediator transforming growth factor (TGF)-*β*_1_ from left ventricular myocardium (a) and relative myocardial TGF-*β*_1_ protein band densities (b) in the groups of SD control, streptozotocin- (STZ-) induced diabetic SD, Zucker Diabetic Fatty (ZDF) lean, and ZDF diabetic rats. Bar graphs represent mean ± SEM. Percent changes between diabetic and corresponding nondiabetic control groups are indicated in both DM models. ^*^: *P* < 0.05 versus the corresponding nondiabetic group.

In Western blots, we detected TGF-*β*_1_ protein in rat myocardium as a single band at 25 kDa. Densitometric analysis of the bands revealed a significant increase in relative TGF-*β*_1_ protein content in myocardium from type-1 diabetic rats in comparison with the corresponding controls. In contrast, significantly decreased TGF-*β*_1_ protein expression could be detected in the type-2 DM model (Figure 5).

## 4. Discussion

In this study, a comparative investigation of functional, histological, and molecular changes in the heart of type-1 and type-2 diabetic rats was performed. The reported differences in cardiac performance between the two diabetes models were accompanied by an altered pattern of myocardial gene and protein expression and markers of nitrooxidative stress, apoptosis, cardiomyocyte hypertrophy, and fibrotic remodelling of the myocardium. This is the first study reporting characteristic differences on the cellular and subcellular level between rat models of type-1 and type-2 diabetic cardiomyopathy.

In spite of the clinical importance of diabetic cardiomyopathy as a distinct disease entity, the cellular and molecular pathomechanisms triggering the adverse changes in diabetic myocardial structure and function have not been completely understood. Oxidative imbalance and increased nitrooxidative stress, cardiomyocyte apoptosis, and hypertrophy, as well as myocardial fibrosis, have been suggested to be the key pathophysiological features of development and progression of diabetic cardiac complications [9].

Immunohistochemical staining against nitrotyrosine (NT), a marker for nitrooxidative stress, clearly showed increased NT-immunoreactivity in the diabetic myocardium in both diabetes models, which is in line with previous data [13, 14]. According to the semiquantitative analysis of the staining, more pronounced nitrooxidative stress could be documented in the type-1 DM model, when compared with type-2 DM. The compensatory overexpression of endogenous antioxidant systems (such as the SOD-catalase and glutathione systems or thioredoxin) further confirmed a more significant increase of nitrooxidative stress in the myocardium of our type-1 diabetic animals when compared with type-2 DM. Increased nitrooxidative stress can result in triggering pathways of apoptosis, ventricular hypertrophy, and fibrotic remodelling [9].

Dysregulation of apoptosis, a programmed form of cell death, is implicated in several cardiovascular pathologies, including diabetic cardiomyopathy. Increasing body of evidence from both clinical and experimental observations suggests that progressive loss of cardiomyocytes via apoptosis plays an important causal role in the development of diabetic cardiomyopathy [8, 9, 15]. Increased cardiomyocyte apoptosis is involved in the process of transition from compensated to decompensated ventricular dysfunction in the diabetic heart [16] as dead cardiomyocytes are replaced by extracellular matrix components, which leads to the development of collagen deposition and myocardial fibrosis [17, 18]. TUNEL assay is a widely used method for assessing DNA breaks and DNA fragmentation in various cells and thus it has been proposed for detection of cells undergoing apoptosis [19]. Our current results regarding TUNEL-positive cardiomyocyte nuclei (Figures 2 and 3(b)) as well as myocardial expression of the proapoptotic mediator caspase-12 (Figure 4) show increased DNA fragmentation and cardiomyocyte apoptosis in diabetic animals, more remarkably in the type-1 DM model. Correspondingly, previous studies reported diabetes-associated increase in TUNEL positive cardiomyocyte cell nuclei both in type-1 [15] and type-2 diabetic rats [20], but a direct comparison of the two DM models was not available yet.

Several markers of myocardial hypertrophy have been investigated in our study. Cardiomyocyte width showed a significant increase in diabetic rats which was comparable in both models (Figure 3(a)). An increasing tendency in heart weight/body weight ratio was observed in type-2 diabetic rats, which reached the level of statistical significance only in the type-1 DM model; however, this index is severely influenced by marked body weight changes (that were more pronounced in the type-1 DM model).

Molecular mechanisms of the hypertrophic transcriptional program include the transient activation of immediate-early genes that encode transcription factors such as c-jun and c-fos [21, 22]. These early genes were significantly upregulated only in type-1 DM (Figure 4). c-jun and c-fos have been described to activate the fetal gene program (transcription of genes such as *β*-MHC or ANF), which play a key role in adaptive hypertrophy and also in maladaptive changes of cardiomyocytes, and have been widely used as markers of pathological hypertrophy [23]. Decreased expression of *α*-MHC and increase in the fetal type *β*-MHC, as molecular markers of pathological hypertrophy, were present in both DM models, being more prominent in type-1 DM (Figure 4). The rate of myocardial overexpression of the hypertrophy marker ANF was found to be comparable in the two DM models (Figure 4). These results clearly reflect the existence of myocardial hypertrophy associated with diabetes in type-1 and also in type-2 DM models and are in correspondence with previous data [9, 24, 25].

Fibrotic remodelling of the myocardium has been reported to be another key pathophysiological feature in diabetic cardiomyopathy. Profibrotic signalling and interstitial collagen deposition are triggered by overproduction of reactive oxygen and nitrogen species (ROS/RNS) [9] and/or could be a consequence of apoptotic cardiomyocyte death (replacement fibrosis). A more pronounced increase in nitrooxidative stress and apoptosis, along with markedly elevated myocardial content of the profibrotic protein mediator TGF-*β*_1_ (Figure 5), and significantly higher histopathological fibrosis score values (Figure 3(d)) have been demonstrated in type-1 diabetic rats (fibrosis +~260%) when compared to ZDF rats with type-2 DM (fibrosis +~45%). Although the existence of myocardial fibrosis in STZ-induced type-1 DM has been well recognized and widely described [9, 25, 26], there are controversial data regarding the ZDF type-2 DM model. Marsh et al. reported no myocardial collagen deposition in ZDF diabetic animals at 14 weeks of age [27]; other studies demonstrated only perivascular but not interstitial myocardial fibrosis [24] at the age of 19 weeks, while a robust fibrotic remodelling of the left ventricle has been described in 45-week-old ZDF rats [28]. Differences regarding the age of the animals and thus different diabetes duration could be the reason for these discrepancies. Our present results showing ~45% increase in myocardial interstitial fibrosis score in 30–32-week-old ZDF diabetic rats (Figure 3(d)) are in accordance with these literature data. Interestingly, a much shorter diabetes duration (8 weeks) leads to a more severe myocardial fibrosis in the type-1 compared to the type-2 DM model (30–32 weeks of age corresponds to 23–25 weeks of diabetes duration). Although the severity of DM (degree of hyperglycemia) was found to be the same at the end of our investigations (Table 2), pathophysiological/metabolic differences (insulinopenia versus hyperinsulinaemia and insulin resistance in the first phase) and the different time course of DM between the two types of the disease might explain these characteristics. In accordance with previous observations [28] increased fibrosis score values of the Masson's trichrome staining in the diabetic myocardium were associated with depressed mRNA expression of collagens 1 and 3 (Figure 4), suggesting that altered rate of collagen degradation rather than enhanced synthesis is responsible for the observed interstitial fibrosis.

Diabetic cardiomyopathy is associated with changes in the expression and bioavailability of vasoactive factors released from the endothelium of coronary microvasculature, including upregulation of endothelin-1 (ET-1) and downregulation of nitric oxide (NO) [29]. In accordance with these observations we detected a marked increase in the expression of ET-1 in diabetic heart of both DM models (more pronounced in type-1 DM) along with decreased eNOS expression only in animals with type-1 diabetes (Figure 4). These results suggest more severe cardiac microvascular abnormalities in type-1 versus type-2 DM. Increased ET-1 levels in DM have been associated with the development of myocardial fibrotic remodeling through the accumulation of fibroblasts, mediated by the ET-1-induced endothelial-to-mesenchymal transition process [30]. This mechanism might be reflected by the above discussed corresponding differences in the severity of myocardial fibrosis between the two DM models.

The functional alterations described in the diabetic heart are closely associated with molecular and histopathological evidence of cardiomyocyte hypertrophy, apoptosis, and fibrosis as discussed above [9]. The contributing components of these three major features to diastolic (heart failure with preserved ejection fraction, HFPEF) versus systolic dysfunction may be somewhat different. Both cardiomyocyte hypertrophy and cardiac fibrosis are evident in HFPEF, but the latter—along with increased cardiomyocyte apoptosis—is reported to be more prominent in systolic heart failure [9, 17, 18]. In contrast to diabetes-associated HFPEF, myocardial systolic dysfunction in DM may have a greater reliance on cardiomyocyte-generated ROS, cardiomyocyte apoptosis and subsequent extracellular matrix deposition, and fibrotic remodelling of the myocardium [17, 31]. Although not investigated in the present study, additional molecular mechanisms (such as advanced glycation end products (AGE) deposition, collagen cross-linking, and oxidative modifications of sarcomeric proteins) might also contribute to the development of diastolic dysfunction in diabetic animals.

According to the above discussed pathophysiological changes, our previously published [6] and current results on in vivo cardiac function (LV P-V analysis, Figure 1) showed corresponding differences in the characteristics of type-1 and type-2 diabetic cardiac dysfunction. A marked impairment of LV contractility and systolic dysfunction (decreased EF and slope of ESPVR) could be demonstrated only in type-1 DM, which was accompanied by severe nitrooxidative stress, cardiomyocyte apoptosis, and myocardial fibrosis. In contrast to the type-1 DM model, these pathological features were significantly less pronounced or absent, while cardiomyocyte hypertrophy was comparable in type-2 DM. ZDF type-2 diabetic animals showed a marked increase in diastolic stiffness (increased LVEDP and slope of EDPVR) without impairment of systolic performance of the heart (characteristic for HFPEF). We hypothesize that pathophysiological differences in the disease development, severity, and progression between the two different types of DM could be the key factors in the observed functional and molecular characteristics of diabetic cardiomyopathy in type-1 versus type-2 DM.

## 5. Conclusions

In the present study we have provided for the first time a direct comparison of type-1 and type-2 diabetic cardiomyopathy in widely used rodent models. We have shown characteristic functional differences between the two models by LV P-V analysis and revealed the underlying mechanisms: an altered pattern of key pathophysiological features (nitrooxidative stress, cardiomyocyte apoptosis and hypertrophy, and myocardial fibrosis) in the diabetic heart has been observed. Our current results help to understand the pathomechanisms of diabetic cardiomyopathy in type-1 and type-2 DM and can serve as a basis for further experimental research.

## Figures and Tables

**Table 1:** Identification numbers of TaqMan Gene Expression Assays and sequence for the forward (F) and reverse (R) primers (from 5′ to 3′) and Universal Probe Library (UPL) probes used in quantitative real time PCR.

Gene target	TaqMan Gene Expression Assay ID	
Catalase	Rn00560930_m1	
GAPDH	Rn01775763_g1	
GSR	Rn01482159_m1	
SOD2	Rn00690587_g1	
Thioredoxin	Rn00587437_m1	

Gene target	Primer sequence	UPL probes

ANF	F: 5′-CAACACAGATCTGATGGATTTCA-3′ R: 5′-CGCTTCATCGGTCTGCTC-3′	***65***
*α*-MHC	F: 5′-GGAGGTGGAGAAGCTGGAA-3′ R: 5′-ATCTTGCCCTCCTCATGCT-3′	***65***
*β*-actin	F: 5′-CTAAGGCCAACCGTGAAAAG-3′ R: 5′-TACATGGCTGGGGTGTTGA-3′	***115***
*β*-MHC	F: 5′-GCTGCAGAAGAAGCTCAAAGA-3′ R: 5′-GCAGCTTCTCCACCTTGG-3′	***65***
Caspase-12	F: 5′-TGGATACTCAGTGGTGATAAAGGA-3′ R: 5′-ACGGCCAGCAAACTTCATTA-3′	***94***
Collagen-1	F: 5′-CTGGCAACCTCAAGAAGTCC-3′ R: 5′-CAAGTTCCGGTGTGACTCG-3′	***65***
Collagen-3	F: 5′-CCTGTTGGTCCATCTGGAAA-3′ R: 5′-GACCTTGGGGACCAGGAG-3′	***82***
c-fos	F: 5′-CCTGCAAGATCCCCAATG-3′ R: 5′-AGTCAAGTCCAGGGAGGTCA-3′	***115***
c-jun	F: 5′-TACCGGCCAGCAACT TTC-3′ R: 5′-TAGGCGCAGAAGAGGTTTTG-3′	***115***
eNOS	F: 5′-TGACCCTCACCGATACAACA-3′ R: 5′-CGGGTGTCTAGATCCATGC-3′	***5***
ET-1	F: 5′-TGTCTACTTCTGCCACCTGGA-3′ R: 5′-CCTAGTCCATACGGGACGAC-3′	***115***
GAPDH	F: 5′-AGCTGGTCATCAATGGGAAA-3′ R: 5′-CGGCAGGTCCTTCTCTATCA-3′	***9***
SOD1	F: 5′-GGTCCAGCGGATGAAGAG-3′ R: 5′-GGACACATTGGCCACACC-3′	***5***

Atrial natriuretic factor (ANF), alpha-myosin heavy chain (*α*-MHC), beta-myosin heavy chain (*β*-MHC), endothelial nitric oxide synthase (eNOS), endothelin-1 (ET-1), glyceraldehyde-3-phosphate dehydrogenase (GAPDH), glutathione reductase (GSR), and superoxide dismutase 1 (SOD1) and 2 (SOD2).

**Table 2:** Blood and urine glucose, body weight, and heart weight to body weight ratio.

	Control SD	STZ-diabetic SD	ZDF lean	ZDF diabetic
Blood glucose (mmol/L)	6.54 ± 0.16	26.08 ± 0.87^*^	6.28 ± 0.29	25.99 ± 2.08^*^
Urine glucose (qualitative)	Negative	Positive	Negative	Positive
Body weight (g)	431.3 ± 5.9	330.0 ± 10.5^*^	440.0 ± 4.8	353.3 ± 9.5^*^
HW/BW (g/kg)	2.92 ± 0.06	3.51 ± 0.14^*^	3.46 ± 0.05	3.74 ± 0.23

Blood and urine glucose, body weight, and heart weight to body weight ratio (HW/BW) values are shown in the groups of control Sprague-Dawley (SD), streptozotocin- (STZ-) diabetic SD, ZDF lean, and ZDF diabetic rats. Values are means ± SEM. ^*^ *P* < 0.05 versus corresponding nondiabetic group.
